# The Ginsenoside 20-O-β-D-Glucopyranosyl-20(S)-Protopanaxadiol Induces Autophagy and Apoptosis in Human Melanoma via AMPK/JNK Phosphorylation

**DOI:** 10.1371/journal.pone.0104305

**Published:** 2014-08-19

**Authors:** Soouk Kang, Jong-Eun Kim, Nu Ry Song, Sung Keun Jung, Mee Hyun Lee, Jun Seong Park, Myeong-Hun Yeom, Ann M. Bode, Zigang Dong, Ki Won Lee

**Affiliations:** 1 WCU Biomodulation Major, Department of Agricultural Biotechnology and Center for Food and Bioconvergence, Seoul National University, Seoul, Republic of Korea; 2 The Hormel Institute, University of Minnesota, Austin, Minnesota, United States of America; 3 Advanced Institutes of Convergence Technology, Seoul National University, Suwon, Republic of Korea; 4 Functional Food Resources Research Group, Korea Food Research Institute, Seongnam, Republic of Korea; 5 Skin Research Institute, Amorepacific Corporation R&D Center, Yongin, Republic of Korea; 6 Research Institute of Bio Food Industry, Institute of Green Bio Science and Technology, Seoul National University, Pyeongchang, Republic of Korea; University of Colorado Denver, United States of America

## Abstract

Studies have shown that a major metabolite of the red ginseng ginsenoside Rb1, called 20-O-β-D-glucopyranosyl-20(S)-protopanaxadiol (GPD), exhibits anticancer properties. However, the chemotherapeutic effects and molecular mechanisms behind GPD action in human melanoma have not been previously investigated. Here we report the anticancer activity of GPD and its mechanism of action in melanoma cells. GPD, but not its parent compound Rb1, inhibited melanoma cell proliferation in a dose-dependent manner. Further investigation revealed that GPD treatment achieved this inhibition through the induction of autophagy and apoptosis, while Rb1 failed to show significant effect at the same concentrations. The inhibitory effect of GPD appears to be mediated through the induction of AMPK and the subsequent attenuation of mTOR phosphorylation. In addition, GPD activated c-Jun by inducing JNK phosphorylation. Our findings suggest that GPD suppresses melanoma growth by inducing autophagic cell death and apoptosis via AMPK/JNK pathway activation. GPD therefore has the potential to be developed as a chemotherapeutic agent for the treatment of human melanoma.

## Introduction

Malignant melanoma is one of the most aggressive forms of cancer, and advanced melanoma has a very poor prognosis. Every year in the US, more than 75,000 people are diagnosed with melanoma, of which 10,000 patients will die of the disease [1]. The development of more potent therapeutic strategies with high specificity for signaling pathways that are dysregulated in melanoma are urgently needed [2]. Mutations of the oncogene B-Raf are a common occurrence in melanoma [3] and lead to hyperactivation of the RAF/MEK/ERK signaling cascade, thereby inhibiting LKB1/AMPK activity, which would normally attenuate proliferation [4]. Although the inhibition of B-Raf is a common therapeutic strategy, acquired resistance to such inhibitors frequently occurs, which greatly reduces further options for successful treatment. To counteract this obstacle, combination therapies with B-Raf/MEK inhibitors together with compounds that can activate the AMPK pathway have been suggested. This double-edged approach may be an effective weapon against melanoma, however, its proof-of-concept has not been extensively demonstrated [5], [6].

Autophagy is a complex process that enables cells to recycle cellular components during times of stress. In certain situations, its activation can function as a tumor suppressor by blocking pathways that lead to cancer cell proliferation [7]. The knockdown of autophagic regulators including Beclin-1 and Atg 5 has been found to increase tumor incidence, although the distinct mechanisms behind the phenomena are not fully understood [7]–[9]. The AMP-activated protein kinase (AMPK) is a key regulator of energy metabolism and activates autophagy through its ability to impair mTORC1 signaling [10], [11]. AMPK is composed of three subunits, involving a catalytic subunit (α subunit) and two regulatory subunits (β and γ subunits). The phosphorylation of AMPK at Thr172 (located on the α subunit) is essential for its full activation [4], and once phosphorylated, the AMPK complex functions as a metabolic checkpoint. Tumor cells must overcome this checkpoint in order to proliferate, and the failure to do so may result in apoptosis [12]. In addition, the JNK pathway has also been implicated to play a major role in autophagy as well as apoptosis of cancer cells [13]. JNK signaling is known to be frequently dysregulated in melanoma cells [14], while in chronic myelogeous leukemia cells, it has been reported that AMPK activation is mediated by JNK-p62 expression, leading to autophagic cell death [15].

**Figure 1:**
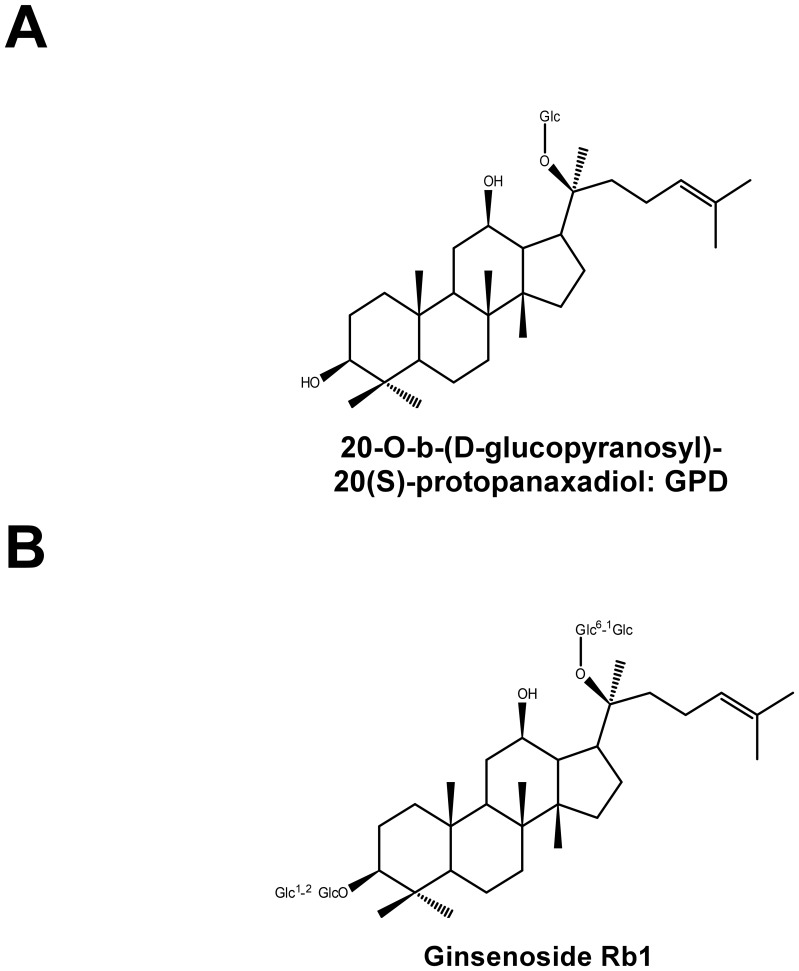
Chemical structures of GPD (A) and ginsenoside Rb1 (B).

In East Asia, ginseng has been employed as a traditional medicine for a variety of ailments for more than 5,000 years [16], [17]. Major pharmacological effects of ginseng extracts include anti-neoplastic, anti-diabetic, neuroprotective, cardioprotective and immunological functions [17]–[19]. Ginsenosides are the major bioactive compounds found in ginseng and represent a diverse group of steroidal saponins. More than twenty ginsenosides have been isolated and identified, while novel structures continue to be reported. The two major sub-types of ginsenosides have been termed protopanaxadiols and protophanaxatriols, which after ingestion can give rise to novel metabolites in the body [18], [19]. 20-O-β-D-glucopyranosyl-20(S)-protopanaxadiol (GPD) (Figure 1A) is a major metabolite of several protopanaxadiol-type ginsenosides including Rb1 (Figure 1B), Rb2 and Rc. GPD is mainly produced as a byproduct of processing by human intestinal bacteria, and is rapidly absorbed into the blood [20], [21]. It has been reported to possess anti-diabetic [22]–[24], anti-inflammatory [25]–[27] and anti-cancer [28], [29] effects. Although the parent compound, ginsenoside Rb1 has also been reported to exhibit anticancer activity [30], [31], a barrier to the efficacy of Rb1 is its limited cellular uptake ratio which limits its bioavailability *in vivo* [29], [32].

Our study is the first to demonstrate the therapeutic potential of GPD and associated signaling mechanisms involved in the induction of autophagy and apoptosis in human melanoma. We report that GPD induces AMPK/JNK activation, leading to the inhibition of malignant cell growth by increasing autophagic and apoptotic processes.

## Materials and Methods

### Chemicals and reagents

GPD was obtained from the Ambo Institute (Seoul, Republic of Korea). The antibody against LC3 was purchased from ABGENT Inc. (San Diego, CA), while antibodies against phosphorylated AMPKα (Thr172), total AMPKα, phosphorylated c-Jun NH2-terminal kinases (JNKs; Thr183/Tyr185), total JNK, Beclin-1, phosphorylated Bcl-2, cytochrome C, PARP, caspase-3, 7, 9, phosphorylated ATF2, ATF, phosphorylated c-Jun (Ser73), total c-Jun, phosphorylated mTOR (Ser2448) and total mTOR were purchased from Cell Signaling Technology (Danvers, MA). The antibodies against p62/SQSTM1, Bcl-2, α-tubulin and β-actin were purchased from Santa Cruz Biotechnology, Inc. (Santa Cruz, CA, USA). The BCA protein assay kit and mitochondrial isolation kit for cultured cells were obtained from Thermo Scientific (Rockford, IL). Puromycin was purchased from InvivoGen (San Diego, CA). 5-bromo-2′-deoxyuridine and anti-bromodeoxyuridine were purchased from Roche Applied Science (Indianapolis, IN).

### Cell culture and transfection

SK-MEL-28 cells were obtained from ATCC (Manassas, VA). The cells were cultured in Dulbecco's Modified Eagle's Medium (DMEM) supplemented with 10% fetal bovine serum, antibiotics and 1× MEM non-essential amino acids in a 5% CO_2_ incubator at 37°C. For transfection experiments, jetPEI (Qbiogen, Inc.) transfection reagent was used, following the manufacturer's instructions.

### Bromodeoxyuridine (BrdU) assay

The BrdU assay was used to evaluate the effects of GPD on SK-MEL-28 cell proliferation. Cells (1.2×10^5^) were seeded in 60 mm dishes and treated with 20 µM, 40 µM and 60 µM of GPD or 60 µM of ginsenoside Rb1 for 48 h. Cells were labeled by incubation with 10 µM BrdU for 30 min before harvesting. The cells were harvested and fixed with 70% ethanol at 4°C for at least 30 min. After denaturing cellular DNA, cells were suspended in 100 µL solution containing 2 µg/mL anti-BrdU antibody diluted in PBS containing 0.1% BSA and incubated for 30 min at room temperature. Cells were washed with PBS twice and re-suspended in 100 µL of diluted goat anti-mouse IgG-FITC. Cells were analyzed on a FACS Calibur flow cytometer (BD Biosciences) and the ratio against untreated control of BrdU labeled cells was measured.

### Anchorage-independent cell growth assay

Cells (8×10^3^ per well) were suspended in BME (1 mL with 10% FBS and 0.33% agar) and plated over a layer of solidified BME/10% FBS/0.5% agar (3 ml) with various concentrations of GPD or 40 µM of Rb1. The cultures were maintained at 37°C in a 5% CO_2_ incubator for up to 7 days, and the cell colonies were counted under a microscope with the aid of Image-Pro Plus software (version 6.2; Media Cybernetics).

### Immunofluorescence and confocal microscopy

SK-MEL-28 cells were stably transfected with GFP-LC plasmids and selected using 2 µg/ml of puromycin. Cells (5×10^3^) were seeded in 4-chamber polystyrene vessel tissue culture glass slides (BD Biosciences, Inc., Sparks, MD), and treated with 20, 40 µM GPD or 40 µM Rb1 for 72 h in a 37°C incubator. Cells were fixed with 4% formalin for 15 min at room temperature in the dark. After washing twice for 10 min each with PBS containing Ca+ and Mg+, cells were blocked with PBS/0.02% Tween20/1% BSA in a 37°C incubator for 1 h. Cells were then incubated with a 1∶100 dilution of p62/SQSTM1 mouse antibody at 37°C for 1 hr. After washing three times for 5 min each with PBS/0.02% Tween20/1% BSA, cells were incubated with a 1∶1000 dilution of Alexa Fluor 568 goat anti-mouse antibody for 1 h in the dark. Co-localization of proteins was observed by laser scanning confocal microscopy (NIKON C1si Confocal Spectral Imaging System, NIKON Instruments Co.) using a CFI Plan Fluor 40× oil objective and then analyzed using the EZ-C1 (v3.90) software program.

### DNA fragmentation

SK-MEL-28 cells (1×10^6^) were seeded and incubated for 24 h. Cells were then treated with GPD (10, 20, 40 µM) or Rb1 (40 µM) for 72 hr. The cells were disrupted by adding lysis buffer (10 mM Tris-HCl, pH 8.0, 10 mM EDTA, 0.5% Triton X 100) with 5 µl of proteinase K and incubated at 50°C for 4 h, and centrifuged at 12,000× g for 30 min at 4°C. DNA was extracted twice with phenol∶chloroform∶isopropyl alcohol (25∶24∶1). The supernatant fraction containing fragmented DNA was mixed with 0.5 ml of isopropanol at room temperature for 10 min and centrifuged at 12,000 g for 10 min at 4°C. The DNA pellet was washed with 75% ethanol and resuspended in Tris-HCl (pH 8.0) with 100 µg/ml RNAse for 2 h. DNA fragments were separated using 1.8% agarose gel electrophoresis, stained with ethidium bromide, and photographed under UV light.

### Annexin V staining

Apoptosis was performed using the annexin V-FITC apoptosis detection kit (MBL International Corp., Watertown, MA). The ratio of total apoptosis was measured in SK-MEL-28 cells that were treated with various concentrations of GPD or 40 µM of Rb1 for 48 h. The cells were harvested and washed with PBS and incubated for 5 min at room temperature with annexin V-FITC, plus propidium iodide following the protocol instructions. Cells were analyzed on a FACS Calibur flow cytometer (BD Biosciences).

### Western blot analysis

Proteins were resolved by SDS-PAGE and transferred onto polyvinylidene difluoride membranes (Millipore), which were blocked with 5% skim milk and incubated with specific primary antibodies overnight at 4°C. The protein bands were visualized using ECL solution (Thermo Scientific) after hybridization with a horseradish peroxidase–conjugated secondary antibody.

### Immunoprecipitation

Bcl-2 antibody was used for immunoprecipitation of GPD treated SK-MEL-28 cell lysates (1 mg of each). The cell lysates were precleared by incubating with 30 µl agarose A/G beads (50% slurry) for 2 h at 4°C. The beads were removed and 30 µl of fresh agarose A/G beads (50% slurry) and appropriate antibodies were added to the precleared lysates, followed by overnight incubation at 4°C. The beads were washed, mixed with 6× SDS sample buffer, boiled, and then resolved by 15% SDS–PAGE. The protein was analyzed by Western blot as described above.

### Statistical analysis

All quantitative results are expressed as mean values ±SE. Statistically significant differences were obtained by Student's t test. A value of p<0.05 was considered to be statistically significant.

## Results

### GPD inhibits the growth of SK-MEL-28 cells

**Figure 2:**
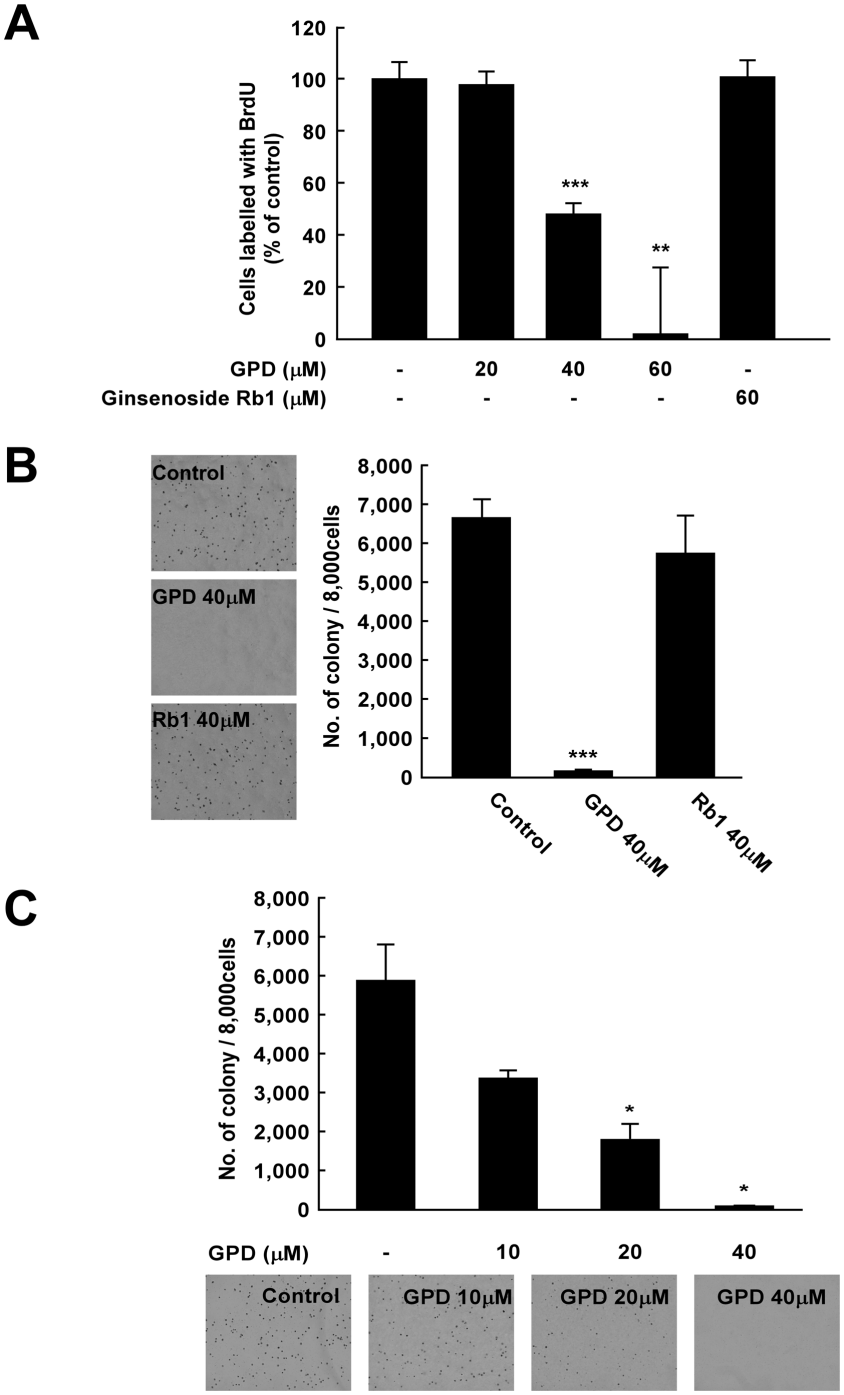
GPD inhibits both anchorage-dependent and -independent growth in SK-MEL-28 human melanoma cells. (A) GPD inhibits the proliferation of SK-MEL-28 cells whereas Rb1 exhibits no significant effect. BrdU-incorporated cells were treated with various concentrations of GPD (20, 40, 60 µM) or Rb1 (60 µM). Results are shown as % of control ±SD (n = 4). The asterisks (**) and (***) indicate statistical significance (p<0.001 and p<0.0001, respectively) compared with the untreated control groups. (B) GPD markedly inhibits the anchorage-independent cell growth of SK-MEL-28 human melanoma cells, while Rb1 does not. A soft agar assay was performed with or without 40 µM of GPD or 40 µM of Rb1 and the number of colonies was counted under a microscope with the aid of Image-Pro Plus software (Version 6.2). Results are shown as means ±SE (n = 3). The asterisks (***) indicates a significant difference (p<0.001) compared with untreated control groups. (**C**) The effect of GPD on anchorage-independent cell growth inhibition is dose-dependent. The soft agar assay was repeated with various concentrations of GPD. Results are shown as means ±SE (n = 3). The asterisk (*) indicates statistical significance (p<0.05).

To determine whether GPD (Figure 1A) can affect melanoma cell growth, we first investigated the effects of GPD and Rb1 on SK-MEL-28 cell proliferation. BrdU assay results showed that GPD suppressed SK-MEL-28 cell growth in a dose-dependent manner, while ginsenoside Rb1 did not exert any effect at 60 µM (Figure 2A). GPD, but not Rb1, also inhibited anchorage-independent cell growth in SK-MEL-28 at 40 µM (Figure 2B). Treatment of GPD at 10, 20, and 40 µM suppressed anchorage-independent cell growth of SK-MEL-28 in a dose-dependent manner (Figure 2C).

### GPD induces autophagy in SK-MEL-28 cells

**Figure 3:**
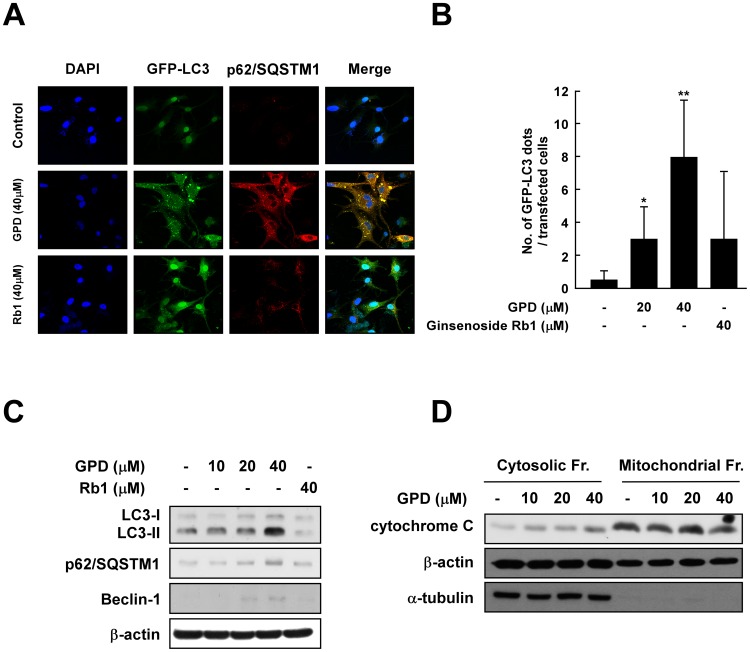
GPD induces autophagic cell death in a dose-dependent manner. (A) GPD induces autophagy in SK-MEL-28 cells whereas Rb1 cannot. GFP-LC3-transfected SK-MEL-28 cells were incubated with or without 40 µM of GPD and compared with Rb1 treatment for 48 h. Cells were fixed with 4% formaldehyde and then incubated with anti-p62/SQSTM1 antibody as described in Materials and Methods. Immunofluorescent staining was observed by confocal microscopy. (B) LC3 aggregations in transfected cells increase in the presence of GPD treatment in a dose-dependent manner. GFP-LC3 aggregations were counted by microscopic (×400) observation of the immunofluorescently stained cells. Results are shown as means ±SD. The asterisks (*) and (**) indicate statistical significance (p<0.05 and p<0.001, respectively) compared with untreated control groups. (C) The expression of LC3-II, p62/SQSTM1 and Beclin-1, which are critical autophagic markers, increased in the presence of GPD treatment, but not Rb1 treatment. Cells were treated with or without various concentration of GPD or 40 µM of Rb1 for 48 h, and protein expression was determined by western blot analysis. (D) GPD increased the translocation of cytochrome c from the mitochondria to the cytosol. Mitochondrial fractionation was performed as described in Materials and Methods.

GPD treatment was found to induce significant alterations in cell morphology, including enlargement of the cytoplasm and generation of membrane vacuoles, phenotypes which have been reported to be representative characteristics of autophagy [10]. Hence, we further investigated whether GPD induces autophagy by studying the marker proteins LC3 and p62/SQSTM1. Immunofluorescence results showed that LC3 aggregations dramatically increased after 40 µM of GPD treatment for 72 h, whereas Rb1 treatment did not exhibit a similar effect (Figure 3A). Additionally, p62/SQSTM1 (an autophagic mediator) protein levels markedly increased and were found to co-localize with LC3 in the cytosol in GPD-treated cells (Figure 3A). Quantification of the GFP-LC3 results by microscopic (×400) observation confirmed that LC3 aggregations increased in GPD-treated cells in a dose-dependent manner, whereas no such observations were apparent for 40 µM ginsenoside Rb1-treated cells (Figure 3B). The prominent autophagic markers LC3–II, p62/SQSTM1 and Beclin-1 were all found to be induced by GPD treatment (Figure 3C). GPD also increased cytochrome c concentrations in the cytosol, concurrent with a decrease in the mitochondrial fraction (Figure 3D).

### GPD induces apoptotic cell death in SK-MEL-28 cells

**Figure 4:**
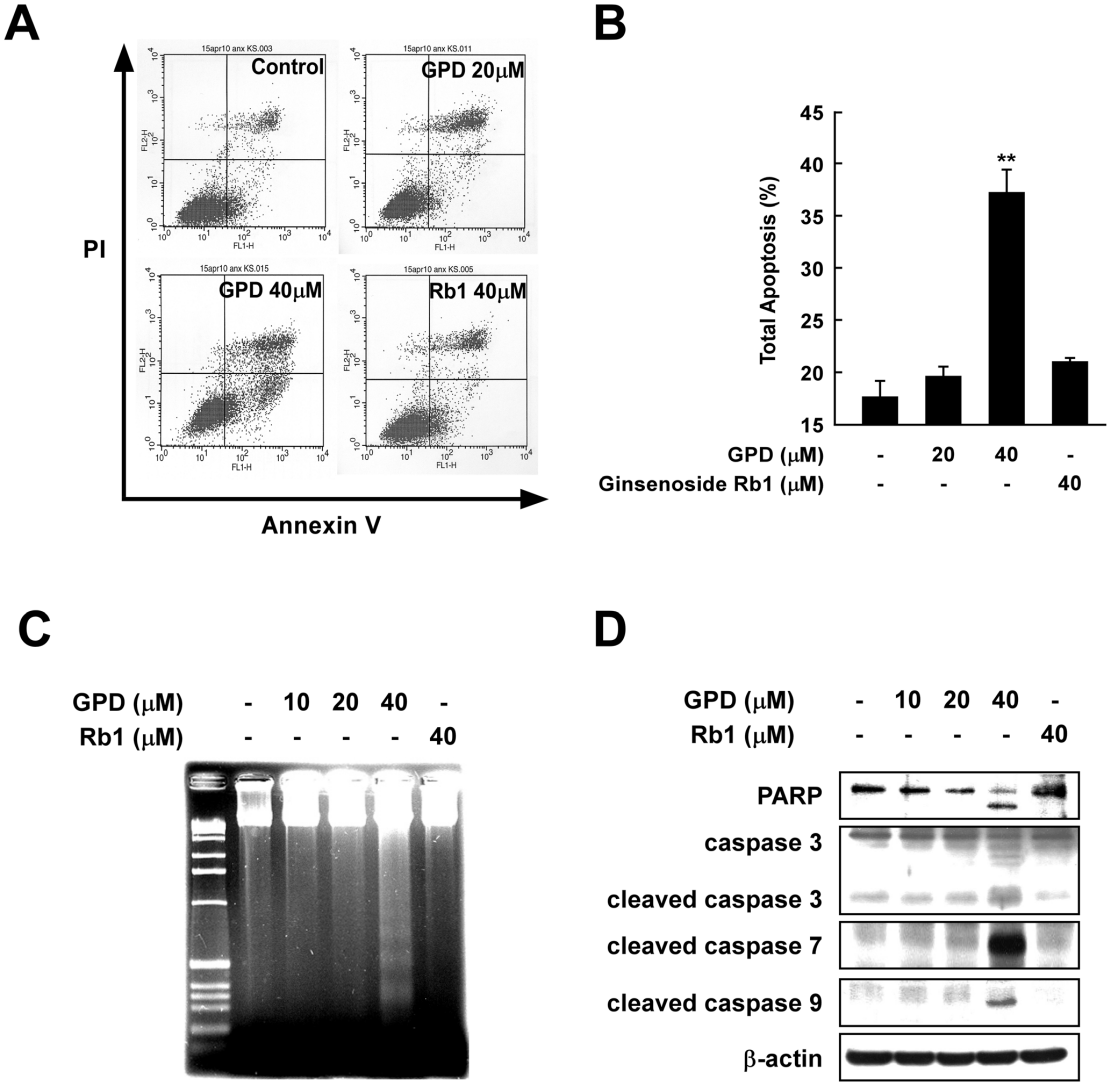
GPD induces apoptosis in SK-MEL-28 human melanoma cells. (A) GPD induces apoptosis in SK-MEL-28 cells. Cells (1.2×10^5^/60 mm dish) were incubated with or without 20 µM, 40 µM of GPD or 40 µM Rb1 for 48 h and apoptotic cells were stained with annexin V and propidium iodide (PI). (B) Apoptosis was determined by Fluorescence Activated Cell Sorting (FACS). The results are shown as mean of total apoptosis % ±SE (n = 3). The asterisk (**) indicates a significant difference (p<0.005) compared with the untreated control group. (C) GPD increases DNA fragmentation in SK-MEL-28 human melanoma cells. Cells were incubated with or without 20 µM, 40 µM of GPD or 40 µM Rb1 for 72 h and fragmented DNA was detected using a fragmentation assay as described in Materials and Methods. (D) The expression of the apoptotic markers cleaved PARP and cleaved caspases 3, 7 and 9, increased after 40 µM of GPD treatment, as confirmed by western blot analysis.

Since the release of cytochrome c from the mitochondria is known to trigger apoptosis, we further investigated apoptotic cell death in SK-MEL-28 cells with GPD treatment. Annexin V and propidium iodide (PI) staining revealed that GPD significantly induced apoptosis in SK-MEL-28 cells (Figure 4A and B). Moreover, GPD, but not ginsenoside Rb1, increased DNA fragmentation (Figure 4C), and also resulted in higher expression of the cleaved (activated) forms of PARP, caspase-3, caspase-7, and caspase-9 (Figure 4D).

### GPD activates the AMPKα and JNK signaling pathways

**Figure 5:**
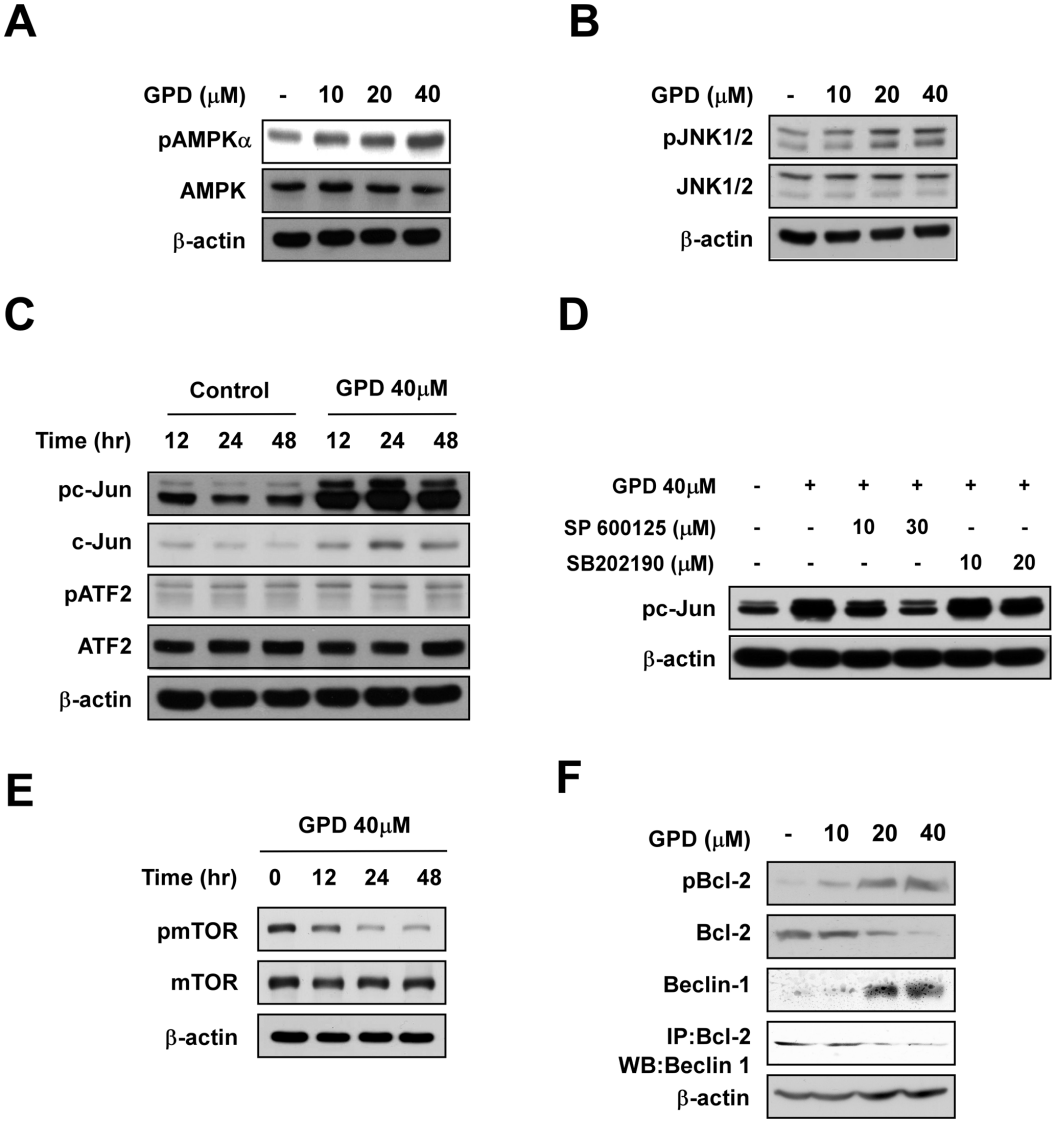
GPD activates the AMPKα and/or JNK pathways in SK-MEL-28 human melanoma cells. (A) Phosphorylation of AMPKα is increased by GPD in a dose-dependent manner. (B) GPD upregulates phosphorylated JNK1/2 in a dose-dependent manner. (C) Levels of phosphorylated c-Jun, a well-known downstream of JNK, peak at 24 h after GPD treatment. (D) The phosphorylation of c-Jun after GPD treatment was dramatically decreased by co-treatment with the JNK inhibitor SP600125, whereas it decreased slightly in the presence of SB202180, a p38 inhibitor. (E) Activated AMPKα negatively regulated the phosphorylation of mTOR in a time-dependent manner. Cells (5×10^5^/100 mm dish) were incubated with various concentrations of GPD for the indicated durations. (F) GPD induces the phosphorylation of Bcl-2 and the dissociation of Beclin-1 from Bcl-2. Western blot analysis was performed using specific antibodies as described in Materials and Methods. β-actin was used to verify equal protein leading.

To investigate the possibility that AMPK was playing a key role in upregulating autophagic cell death [33] and apoptosis induced by GPD, we examined the effects of GPD and ginsenoside Rb1 on the phosphorylation of AMPK in SK-MEL-28 cells. Western blot assay results showed that GPD increased phosphorylation of AMPKα (Figure 5A). GPD also increased the phosphorylation of JNK1/2 (Figure 5B). Moreover, phosphorylation of c-Jun, a well-known downstream substrate of JNK, increased from 12 h to 48 h of GPD treatment, when compared to control (Figure 5C). To confirm whether the phosphorylation of c-Jun was due to JNK activation, cells were incubated with 40 µM of GPD and either the JNK inhibitor SP600125 or the p38 inhibitor SB202180. The western blot results demonstrated that phosphorylated c-Jun after GPD treatment was dramatically decreased only when co-treated with the JNK inhibitor SP600125 (Figure 5D). GPD treatment also attenuated the phosphorylation of mTOR, a negatively regulated downstream substrate of AMPK, in a time-dependent manner (Figure 5E). Finally, we sought to verify if there is a connection between GPD-induced autophagy and apoptosis. Crosstalk between autophagy and apoptosis is mediated by an interaction between Bcl-2 and Beclin-1. Phosphorylation of Bcl-2 by JNK allows dissociation of Bcl-2 from Beclin-1 [34]. We showed that GPD elevated phosphorylation of Bcl-2 although Bcl-2 expression decreased and in turn, induced the disassociation of Bcl-2 from Beclin-1 (Figure 5F). In addition, GPD increased Beclin-1 expression. These results indicate an interconnection between GPD-induced autophagy and apoptosis.

## Discussion

Melanoma remains one of the leading causes of cancer-related deaths [1]. Although the FDA-approved melanoma drug vemurafenib is currently employed in therapeutic settings, overall clinical outcomes have been disappointing due to the emergence of drug resistance [35]. This may be caused by the upregulation of various oncogenic receptor tyrosine kinases, as well as N-RAS and dimerization of aberrantly-spliced B-Raf [36], [37]. Activating mutations of B-Raf have been reported in approximately 60% of melanoma patients [3] with the most frequent mutation being the V600E mutation, the presence of which has been closely linked to a poor prognosis [38]. Drug resistance as a result of B-Raf activating mutations is currently considered one of the most important clinical issues in modern melanoma therapy [39] Therefore, alternative strategies and drugs are urgently needed to overcome this challenge.

Numerous studies have reported that various natural chemicals can be effective at inducing both autophagy and apoptosis [40], and we sought to investigate the possibility that GPD may cause apoptosis in SK-MEL-28 cells. The results showed that GPD, but not Rb1, inhibited cell proliferation and anchorage-independent melanoma cell growth in a dose-dependent manner. Following GPD treatment, we observed changes in cellular morphology and confirmed that this occurred concomitantly with the induction of autophagy. After analyzing mitochondrial and cytoplasmic fractions, we determined that GPD treatment caused the release of cytochrome C into the cytoplasm, a critical step in the apoptotic pathway. Annexin V and DNA fragmentation assay results further confirmed that GPD increased apoptosis. Western blot assay results showed that GPD increased the expression of a plethora of apoptotic proteins including cleaved-PARP, caspase-3, caspase-7, and caspase-9 in SK-MEL-28 cells.

Autophagy is an essential mechanism for maintaining cell homeostasis and involves lysosomal degradation of cytoplasmic components [41]. It functions to allow the recycling of cellular components during times of stress, and it has been found that agents that promote autophagy in malignant cells can inhibit proliferation and induce apoptosis [42]. Starvation or the removal of growth factors in cells induces autophagy, concurrent with the upregulation of regulatory proteins including LC3 and Beclin-1 [43]. The conversion of LC3-I to LC3-II is tightly linked with formation of the autophagosome and is therefore a prominent marker of the process [44]. When applied to melanoma cells, the antidiabetic drug metformin inhibits proliferation through the induction of autophagy, and preferentially induces cleavage of LC3 in malignant cells while leaving normal cells relatively unaffected. Importantly, the inhibition of autophagy decreases the extent of apoptosis, suggesting that apoptosis is a consequence of extended autophagy [45]. Our SQSTM1 western blot assay results showed that GPD upregulated LC3-II, p62/SQSTM1, and Beclin-1 expression and immunofluorescence analysis also revealed that treatment with GPD induced the aggregation of LC3 with its co-factor, p62. Moreover GPD inhibited the anchorage-independent cell growth in PANC-1 pancreatic carcinoma in a dose-dependent manner (Figure S1) which cells possess overactivated C-Raf due to activated K-Ras mutation instead of B-Raf mutation. However, in the current study we could not find the direct target responsible for causing autophagic cell death by GPD. Proteomic or genomic approaches to identify major targets of GPD should be further studied.

The AMPK pathway plays an important regulatory role in the metabolism of both normal and malignant cells, and AMPK activators have been shown to suppress mTOR activity, negatively regulating malignant transformation and cell proliferation in various cancer cell types [4]. Our western blot results showed that GPD increased the phosphorylation of AMPK and JNK in a dose-dependent manner. Activated AMPK negatively regulated the phosphorylation of mTOR, while activated JNK increased the phosphorylation of c-Jun.

Autophagy is also negatively regulated by mTOR signaling, particularly in relation to mTOR Complex 1, which senses nutrient conditions through the actions of GTP-bound Rheb [46]. An important connection exists between mTOR and AMPK. During times of nutrient depletion, the cellular ratio of ATP to AMP decreases, causing the activation of AMPK and leading to the phosphorylation of TSC2 [47]. This provides an explanation for the mechanism behind AMPK-mTOR regulation, as TSC2 is a major inhibitor of mTOR activation.

Bcl-2, an anti-apoptoic protein, is a major regulator of the crosstalk between apoptosis and autophagy. Bcl-2 binds with Beclin-1 and inhibits autophagy. Phosphorylation of Bcl-2 by JNK dissociates Bcl-2 from beclin-1, and free beclin-1 plays a crucial role in inducing autophagy [34]. Therefore, activation of JNK by GPD suggests a link between GPD-induced autophagy and apoptosis. Indeed, we showed that GPD both increased phosphorylation of Bcl-2 and induced subsequent disassociation of Bcl-2 from Beclin-1, which in turn, may increase apoptosis and induce autophagy, respectively. Therefore, Bcl-2 interconnects autophagy and apoptosis induced by GPD.

**Figure 6:**
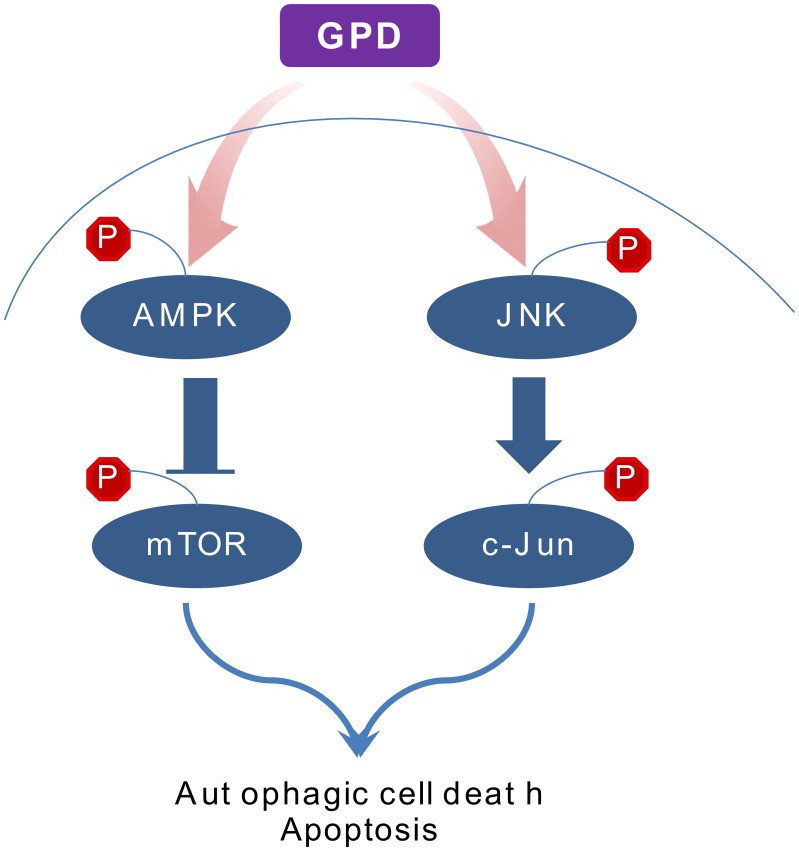
Hypothetical model for the anticancer effect of GPD in human melanoma.

In the current study we have shown that GPD exhibits potent cancer inhibitory effects against human melanoma cells by inducing autophagic cell death and apoptosis. The inhibitory effect of GPD is mediated through AMPK/JNK signaling (Figure 6). These results suggest that GPD is a potential drug candidate for treating melanoma. However, the precise nature of the role of autophagy in melanoma progression remains to be clearly elucidated. Further study is warranted for functional food compounds like GPD, which can manipulate pathways that may halt the progression of malignant growth.

## Supporting Information

Figure S1 **GPD inhibits anchorage-independent cell growth in PANC-1 human pancreatic carcinomas.** Anchorage-independent cell growth in pancreatic carcinoma, PANC-1 cells was inhibited by GPD in a dose-dependent manner. A soft agar assay was performed with or without 10, 20, 40 µM of GPD or 40 µM of Rb1 and cell colonies were counted under a microscope with the aid of Image-Pro Plus software (Version 6). Results are shown as means ±SE (n = 3). The asterisks (*), (**) and (***) indicate statistical significance (p<0.05, p<0.005 and p<0.001, respectively) compared with untreated control groups.
(TIF)
