# Supplementary figures and images for: The Ginsenoside 20-O-β-D-Glucopyranosyl-20(S)-Protopanaxadiol Induces Autophagy and Apoptosis in Human Melanoma via AMPK/JNK Phosphorylation
Source: PLoS One. 2014 Aug 19;9(8):e104305. doi: 10.1371/journal.pone.0104305 (PMC4138097; doi:10.1371/journal.pone.0104305)

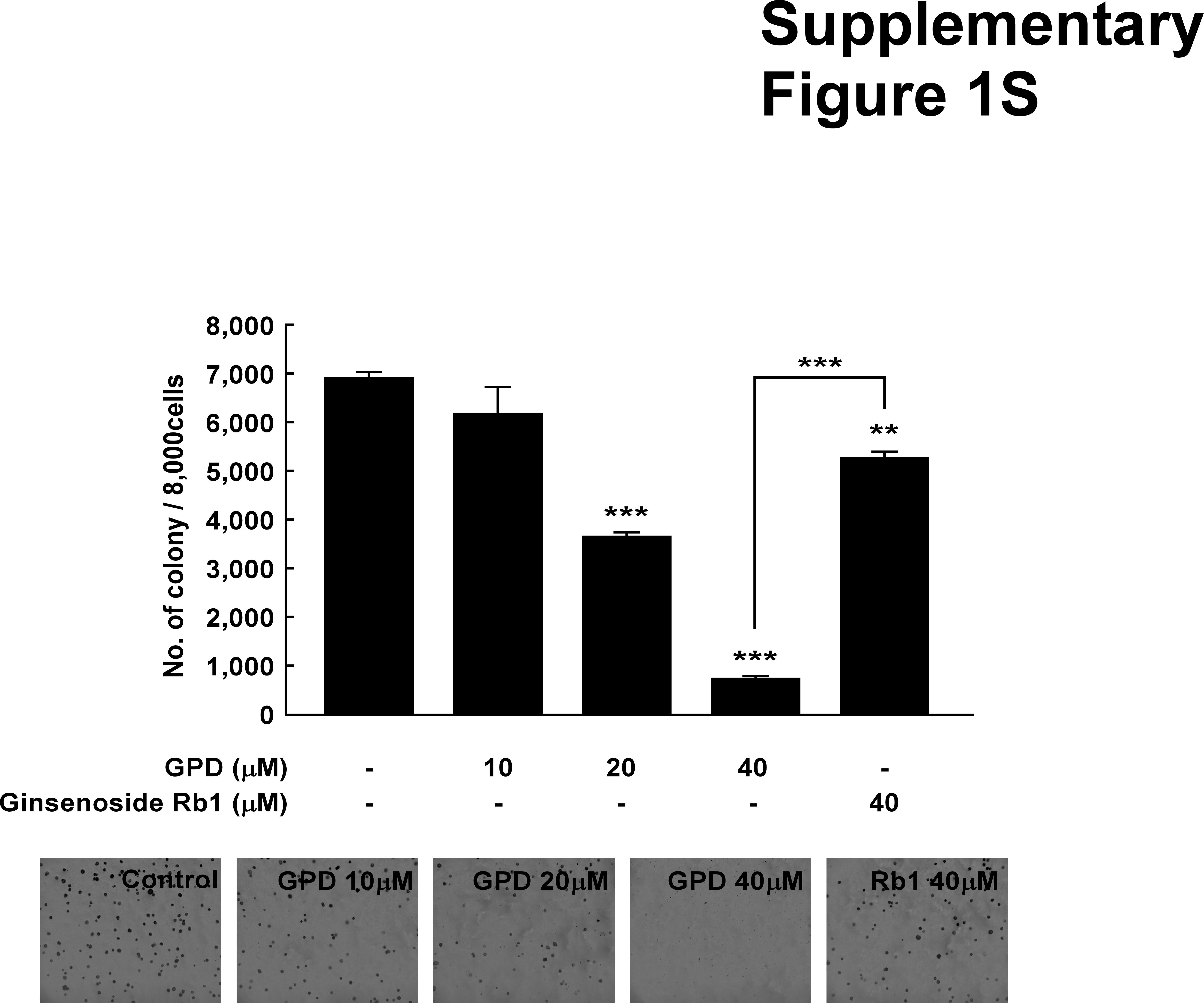

Supplement: Figure S1 — GPD inhibits anchorage-independent cell growth in PANC-1 human pancreatic carcinomas. Anchorage-independent cell growth in pancreatic carcinoma, PANC-1 cells was inhibited by GPD in a dose-dependent manner. A soft agar assay was performed with or without 10, 20, 40 µM of GPD or 40 µM of Rb1 and cell colonies were counted under a microscope with the aid of Image-Pro Plus software (Version 6). Results are shown as means ±SE (n = 3). The asterisks (*), (**) and (***) indicate statistical significance (p<0.05, p<0.005 and p<0.001, respectively) compared with untreated control groups. (TIF) [file pone.0104305.s001.tif]
